# Impact of software tools and kinetic model selection on myocardial blood flow and flow reserve quantitation in ^13^N‐ammonia PET

**DOI:** 10.1002/acm2.70605

**Published:** 2026-05-01

**Authors:** Naochika Akiya, Kenta Miwa, Akira Ando, Noriaki Miyaji, Tensho Yamao, Kaito Wachi, Masaki Masubuchi, Shu Kimura, Reiji Ito, Arata Komatsu, Takuma Horikawa, Kenji Fukushima, Atsutaka Okizaki, Shiro Miura

**Affiliations:** ^1^ Department of Radiological Sciences, Graduate School of Health Sciences Fukushima Medical University Fukushima‐shi Fukushima Japan; ^2^ Department of Radiological Sciences School of Health Sciences Fukushima Medical University Fukushima‐shi Fukushima Japan; ^3^ Division of Diagnostic Radiology Imaging Sapporo Kojinkai Memorial Hospital Sapporo‐shi Hokkaido Japan; ^4^ Fukushima Medical University Aizu Medical Center Hospital Aizuwakamatsu‐shi Fukushima Japan; ^5^ Department of Radiology and Nuclear Medicine Fukushima Medical University Fukushima‐shi Fukushima Japan; ^6^ Department of Radiology Asahikawa Medical University Asahikawa Hokkaido Japan; ^7^ Department of Cardiology Sapporo Kojinkai Memorial Hospital Sapporo‐shi Hokkaido Japan

**Keywords:** ^13^N‐ammonia positron emission tomography, myocardial blood flow, perfusion, tissue compartment model

## Abstract

**Background:**

Quantitative assessment of myocardial perfusion using ^13^N‐ammonia PET with compartmental modeling enables evaluation of myocardial flow reserve (MFR) and prediction of patient prognosis. However, the reliability of these assessments can depend on the analytical methods used for quantitation.

**Purpose:**

The present study aimed to evaluate the variability and agreement of values obtained using three quantitative software tools and to assess the impact of kinetic model selection on myocardial blood flow (MBF) and MFR estimates in a clinical setting.

**Methods:**

We analyzed 100 patients who underwent ^13^N‐ammonia PET/CT, including 60 with normal perfusion and 20, 10, and 10 with single‐, two‐, and three‐vessel disease, respectively. We derived MBF and MFR at global (entire left ventricle) and regional (coronary territories) levels and evaluated five analytical pipelines: SyngoMBF, QPET, and three implementations of PMOD tools (1‐tissue compartment, Hutchins, and UCLA models).

**Results:**

MBF and MFR showed high correlations among the software tools, although stress MBF statistically differed between PMOD and QPET. Correlation coefficients between software tools ranged from 0.81 to 0.91 at the global level, and Bland–Altman analysis demonstrated overall agreement with residual variability. In contrast, MBF and MFR values varied depending on the compartment model. The UCLA model yielded the highest stress MBF and MFR, and correlation coefficients between models ranged from 0.43 to 0.99 at the global level. Although Bland–Altman analysis showed overall agreement, noticeable scatter persisted and the UCLA model exhibited a positive bias.

**Conclusion:**

Quantitative MBF and MFR estimates from ^13^N‐ammonia PET show good overall agreement across commonly used software tools but remain strongly dependent on kinetic model selection. These findings indicate that quantitative results are not directly interchangeable across different software and modeling approaches, underscoring the importance of methodological consistency when interpreting myocardial perfusion PET in clinical practice.

## 1 INTRODUCTION

^13^N‐ammonia PET is appropriate for measuring myocardial perfusion due to the high spatial resolution of images for visual interpretation^1^, ^2^ and the high first‐pass extraction rate of the tracer during initial circulation.^3^ Visual assessment enables evaluation of ischemia severity, infarction, and prognosis,^4^, ^5^, ^6^, ^7^ while combining it with quantitative analysis improves the precision of risk stratification.^8^, ^9^ Quantitative PET is particularly useful for patients with complex coronary artery disease (CAD), such as left main trunk and 3‐vessel disease.^2^, ^9^, ^10^, ^11^ The primary quantitative metrics are myocardial blood flow (MBF), which represents absolute perfusion to the myocardium (mL/g/min) under stress and rest conditions, and myocardial flow reserve (MFR), defined as the ratio of stress to rest MBF.^12^, ^13^

Quantitative analysis follows a standardized workflow. Dynamic PET images are acquired, then activity concentrations are extracted from predefined volumes of interest (VOIs) in the left ventricular (LV) myocardium and blood pool. Kinetic modeling then generates time–activity curves (TACs) and estimates parameters such as transfer constants by fitting these curves. The MBF for ^13^N‐ammonia is derived from these estimated kinetic parameters. Dedicated quantitative software executes these steps in clinical practice.

**FIGURE 1:**
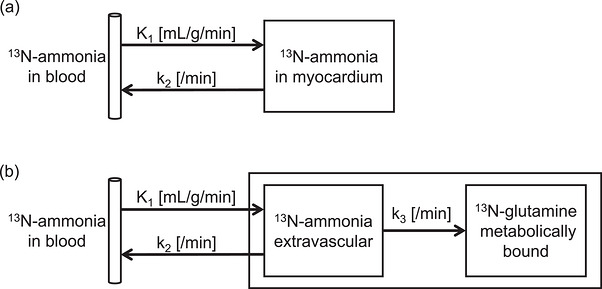
Compartment models used to estimate MBF from ^1^ ^3^N‐ammonia PET. (a) 1‐tissue compartment model (single exchangeable tissue compartment); (b) 2‐tissue compartment model with metabolic‐trapping compartment that encompasses the Hutchins and UCLA variants. Arrows indicate rate constants (*K*1, uptake; *k*2, washout; *k*3, metabolic retention).

Several quantitative software tools are used in clinical and research settings. Differences among implemented algorithms and processing procedures among such tools might affect the consistency of quantitative results. Slomka et al. found good reproducibility and agreement among various types of software used in ^13^N‐ammonia PET quantitation,^14^ whereas others found reduced consistency depending on the software type and version, as well as the clinical heterogeneity of situations.^15^, ^16^, ^17^ Contributing factors include differences in reorientation methods, contour extraction algorithms, definitions of cardiac regions, TAC sampling strategies, and the presence or implementation of motion correction.^18^, ^19^, ^20^ Beyond these technical differences, the choice of compartment model also affects MBF and MFR. Nesterov et al. found high and low ^82^Rb PET reproducibility, respectively, when single or various models were applied.^21^ One‐ and two‐tissue compartment models have been proposed for ^13^N‐ammonia imaging (Figure 1).^22^, ^23^, ^24^ Several kinetic models for ^13^N‐ammonia PET have been described and evaluated in methodological studies. However, the extent to which model selection influences clinically reported MBF and MFR values under standardized analysis conditions has not been systematically examined using contemporary clinical datasets. We aimed to evaluate the variability and agreement of MBF and MFR values assessed using three quantitative software tools and to assess the impact of kinetic model selection on these indices in a clinical setting.

## 2 METHODS

### 2.1 Participants

The Ethics Committee of Sapporo Kojinkai Memorial Hospital approved this study of 100 patients who were assessed by ^13^N‐ammonia PET/CT at Sapporo Kojinkai Memorial Hospital (Approval no: 2024‐6). All patients provided written informed consent to participate in the study, which complied with the ethical principles enshrined in the Declaration of Helsinki (2013 amendment). The patients were retrospectively enrolled between November 2021 and August 2024. Of the 100 patients, 60 had normal myocardial perfusion and 40 had CAD (Table 1). The CAD group was further classified into 20, 10, and 10 patients with single‐, two‐, and three‐vessel disease, respectively, based on the clinical diagnosis.

**TABLE 1:** Characteristics of the patients.

Characteristics	Value
Mean age, years	68.3 ± 12.5
Sex, male/female	60/40
BMI, kg/m^2^	24.7 ± 4.1
Hypertension	60
Dyslipidemia	57
Diabetes	30
Cigarette smokers	19

*Note*: Values are shown as means ± SD or numbers.

### 2.2 Imaging protocol

All images were acquired using a Biograph mCT Flow PET/CT system (Siemens Healthineers, Erlangen, Germany). Three‐dimensional (3D) list‐mode data about stress and rest were collected for 10 min. Low‐dose CT preceded each PET image acquisition for attenuation correction. Pharmacological stress was created by intravenously infusing adenosine triphosphate (ATP) at a rate of 0.16 mg/kg/min for 5 min, with a manual injection of ^13^N‐ammonia (3 MBq/kg) at 3 min, followed by a saline flush. The rest study proceeded using the same protocol 50‐min later. The first 180 s of list‐mode data were re‐binned into 21 frames (of 5 s × 15, 15 s × 5, and 30 s × 1). Images were reconstructed using ordered‐subset expectation maximization (OSEM) with point‐spread‐function (PSF) and time‐of‐flight (TOF) modeling (2 iterations, 21 subsets) and post‐filtered with a 6‐mm Gaussian kernel (FWHM).^25^

### 2.3 Quantitation of MBF and MFR using different software

**FIGURE 2:**
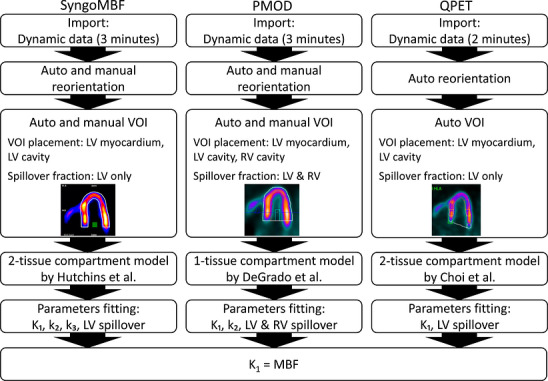
Workflow for MBF quantitation using SyngoMBF, PMOD, and QPET quantitative software tools. Workflow from list‐mode data import to final MBF computation are shown for each tool, which used a distinct compartment model and VOI placement strategy. The VOIs include the LV myocardium, and LV, and RV cavities. Spillover is modeled with either a single LV blood‐pool VOI or dual LV and RV blood‐pool VOIs, according to the algorithm in each tool. LV, left ventricle; MBF, myocardial blood flow; RV, right ventricle; VOI, volume of interest.

The quantitative software packages used to calculate MBF and MFR were SyngoMBF (version VG80B; Siemens Healthineers, Erlangen, Germany), PMOD Cardiac PET Analysis Tool (version 4.404; Bruker, Zurich, Switzerland), and QPET (Cedars‐Sinai Cardiac Suite, Revision 2017.C; Cedars‐Sinai Medical Center, Los Angeles, CA, USA). Figure 2 summarizes the workflow from list‐mode data imported into MBF quantitation for each package.

### 2.4 SyngoMBF

SyngoMBF automatically applies motion correction to later frames, with additional options for severe motion artifacts.^26^, ^27^, ^28^ Myocardial VOIs were automatically drawn on the LV and manually adjusted if necessary. The input‐function VOI (1 × 1 × 1 cm) was positioned on the mitral valve plane. Kinetic modeling followed the 2‐tissue compartment model created by Hutchins et al.,^23^ based on the first 3 min of data. The *K*1, *k*2, *k*3, and vascular volume parameters were estimated using the Levenberg–Marquardt algorithm. A first‐frame‐subtraction method was applied to correct rest TACs for carryover from the stress image.^29^, ^30^ SyngoMBF MBF and MFR outcomes are presented as 17‐segment the American Heart Association (AHA) polar maps.^23^, ^31^ Coronary territories were defined geometrically on polar maps as follows: the left anterior descending artery (LAD) territory encompassed a 150° sector that included the anterior and apex segments of the AHA model and the remaining sectors were assigned clockwise as the left circumflex artery (LCX) and right coronary artery (RCA) territories, each covering 105°.

### 2.5 PMOD

The PMOD tool automatically generated VOIs for the LV myocardium, LV, and right ventricular (RV) cavities, with manual correction as needed. The input‐function VOI comprised six short‐axis slices centered in the LV cavity. Modeling used the 1‐tissue compartment model created by DeGrado et al.^22^ with the first 3 min of data. Parameters were estimated using the Levenberg–Marquardt algorithm to fit the parameters *K*1, *k*2, and spillover fractions from the LV and RV cavities. The spillover fractions represent the contribution of blood‐pool activity from the LV and RV cavities to the measured myocardial activity and were estimated during model fitting. PMOD computed MBF and MFR on the standard 17‐segment AHA model and summarized values for LAD, LCX, and RCA territories.^31^

### 2.6 QPET

QPET applies automatic motion correction to compensate for cardiac displacement during acquisition.^32^ The placement of VOIs was fully automated using algorithms adapted from quantitative gated SPECT for contouring and valve‐plane localization applied to attenuation‐corrected PET images.^33^, ^34^ A cylindrical input‐function VOI (radius and length:1 × 2 cm) was positioned in the LV cavity along the long axis. Kinetic modeling followed the 2‐tissue compartment model created by Choi et al.,^35^ with a fixed distribution volume of 0.8 mL/g. In this model, only the first 2 min of data are used for parameter estimation to minimize the influence of blood metabolites.^24^ The *k*1 and LV‐cavity spillover fraction parameters were estimated using non‐linear least squares. QPET output MBF and MFR on the 17‐segment AHA model and aggregated values for LAD, LCX, and RCA territories.^31^

### 2.7 Quantitation using compartment models

As each software tool implements a fixed kinetic model and does not allow interchangeable models, model comparisons were performed within a single software tool (PMOD). To isolate the effect of kinetic model selection, different compartment models were applied within the same software environment while keeping VOI placement, spillover modeling, and fitting procedures consistent. We applied the PMOD 1‐tissue compartment (1TCM),^22^ Hutchins 2‐tissue,^23^, ^36^ and UCLA 2‐tissue^24^ models to determine the effects of compartment model selection on MBF and MFR. The Hutchins model includes a metabolite correction algorithm described by van den Hoff et al.^36^ Except for the compartment model itself, VOI placements and all other procedures matched those in the *PMOD* section above. The fitting window was 0–3 min for 1TCM and 0–2 min for the Hutchins and UCLA models, respectively. All models incorporated dual spillover from the left and RV cavities. The 1TCM, Hutchins, and UCLA packages contained four, five, and three fitted parameters, respectively. The MBF and MFR were calculated using the 17‐segment AHA model and subdivided into LAD, LCX, and RCA territories.^31^

- 1TCM (DeGrado et al.)^22^
  Fitted parameters (*n* = 4): *K_1_*, *k_2_*, *V_LV_*, *V_RV_*

Input/metabolites: plasma input from LV activity with linear metabolite correction

$$\begin{array}{cc} \frac{dC_{myo}\left(t\right)}{dt} & =k_{1}C_{p}\left(t\right)-k_{2}C_{myo}\left(t\right),\;C_{p}\left(t\right)\\ & =\left(1-mCorr\;*\;t\right)C_{LV}\left(t\right) \end{array}$$

Spillover model:

$$C_{Model}\left(t\right)=\left(1-V_{LV}-V_{RV}\right)C_{myo}\left(t\right)+V_{LV}C_{LV}\left(t\right)+V_{RV}C_{RV}\left(t\right)$$

- Hutchins 2‐tissue model^23^, ^36^

Fitted parameters (*n* = 5): *K_1_*, *k_2_*, *k_3_*, *V_LV_*, *V_RV_*

Input/metabolites: plasma input from LV activity with delayed‐exponential metabolite correction (van den Hoff et al.)

Kinetics:

$$\frac{dC_{1}\left(t\right)}{dt}=K_{1}C_{P}\left(t\right)-\left(k_{2}+k_{3}\right)C_{1}\left(t\right)$$

$$\frac{dC_{2}\left(t\right)}{dt}=k_{3}C_{1}\left(t\right)$$

Metabolite correction

$$C_{P}\left(t\right)=\left\{\begin{array}{c} C_{LV}\left(t\right),\;t\leq t_{0}\\ e^{-\ln2\left(t-t_{0}\right)/T_{1/2}}C_{LV}\left(t\right),\;t>t_{0} \end{array}\right.$$

with delayed time of *t_0 _* = 0.48 min and an effective half‐life (*T_1/2_*) of 6.69 min.

- UCLA 2‐tissue model^24^

Fitted parameters (*n* = 3): *K_1_*, *V_LV_*, *V_RV_*

Constraints/assumptions: *k_2_* and *k_3_* are expressed as functions of *K_1_*; fixed distribution volume (*V_ND_*) of 0.8 mL/g. *V_ND_* represents the distribution volume of free ^13^N‐ammonia in the myocardium and reflects the freely diffusible compartment of the tracer in myocardial tissue.

Kinetics (same state variables as above):

$$\frac{dC_{1}\left(t\right)}{dt}=K_{1}C_{P}\left(t\right)-\left(k_{2}+k_{3}\right)C_{1}\left(t\right)$$

$$\frac{dC_{2}\left(t\right)}{dt}=k_{3}C_{1}\left(t\right)$$

with

$$k_{2}=K_{1}/V_{ND}$$

$$k_{3}=K_{1}\left(1.65e^{1.25/K_{1}}-1\right)$$

Spillover model:

$$\begin{array}{cc} C_{Model}\left(t\right) & =\left(1-V_{LV}-V_{RV}\right)\left(C_{1}\left(t\right)+C_{2}\left(t\right)\right)+V_{LV}C_{LV}\left(t\right)\\ & \;+V_{RV}C_{RV}\left(t\right) \end{array}$$

Common notations: *C_LV_(t)*, *C_RV_(t)*: LV/RV blood‐activity concentrations; *C_P_(t)*: plasma input; *C_myo_(t)*: 1TCM tissue activity; *C_1_(t)*, *C_2_(t)*: exchangeable and metabolically trapped compartments; *V_LV_*, *V_RV_*: spillover fractions; *m_Corr_*, linear metabolite‐correction coefficient.

### 2.8 Statistical analysis

Data were analyzed using GraphPad Prism v10.3.0 (GraphPad Software, San Diego, CA, USA). Both MBF and MFR values are presented as means ± standard deviation (SD). We confirmed that the data were distributed normally before parametric tests. Differences among software packages and compartment models were assessed using one‐way ANOVA, and significant values were followed by Tukey post hoc tests. Values with two‐sided *P* < 0.05 were defined as statistically significant. Pairwise correlations were quantified using Pearson's rho (ρ). Agreement was determined by computing mean bias and the 95% limits of agreement using Bland–Altman analyses of the patients.

## 3 Results

### 3.1 Comparison of MBF and MFR among software packages

Table 2 shows global stress MBF, rest MBF, and MFR (means ± SD) for SyngoMBF, PMOD, and QPET tools. Stress MBF differs among software (one‐way ANOVA, *P* < 0.05). Tukey tests revealed a significant difference between PMOD and QPET for stress MBF, whereas other pairwise differences were not significant. Stratified analyses showed similar trends between groups, with significant differences in stress MBF and MFR observed in the Normal group, primarily between PMOD and QPET (Table S1).

**TABLE 2:** Global stress MBF, rest MBF, and MFR (mean ± SD) results generated using SyngoMBF, PMOD, and QPET software tools.

	SyngoMBF	PMOD	QPET	*p* value
Stress MBF (mL/g/min)	2.42 ± 0.80	2.33 ± 0.71	2.59 ± 0.75	<0.05
Rest MBF (mL/g/min)	0.93 ± 0.20	0.90 ± 0.17	0.93 ± 0.24	0.55
MFR	2.69 ± 0.88	2.62 ± 0.80	2.91 ± 0.97	0.06

**FIGURE 3:**
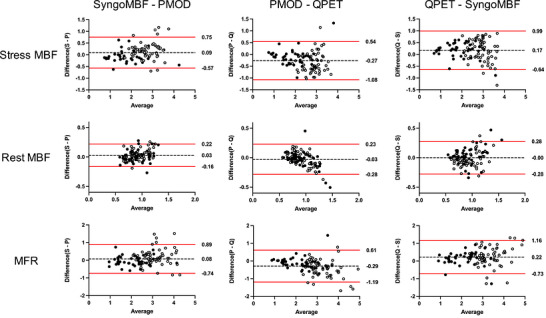
Bland–Altman plots at the global level comparing stress MBF, rest MBF, and MFR between software pairs. The dashed lines indicate the mean difference, and the red solid lines represent the 95% limits of agreement. Open circles represent the Normal group, and filled circles represent the CAD group. S, SyngoMBF; P, PMOD; Q, QPET.

Table 3 shows Pearson correlation coefficients (ρ) for MBF and MFR among the software tools at the global and regional (LAD, LCX, RCA) levels. Correlations were high for all software pairs at both levels. The regional analysis showed that ρ tended to be lower in the RCA territory than in LAD and LCX. Stratified analyses showed overall positive correlations, although relatively lower correlation coefficients for stress MBF were observed in the normal group between PMOD and QPET, as well as between QPET and SyngoMBF (Table S2). Figure 3 shows Bland–Altman plots comparing software tools at the global level, with the Normal and CAD groups indicated by open and filled circles, respectively. Most values were within the 95% limits of agreement, and the degree of variability was similar between the two groups, indicating consistent results among all paired software tools.

**TABLE 3:** Pearson correlation coefficients (ρ) for MBF and MFR among software at the global and regional (LAD, LCX, RCA) levels.

		SyngoMBF–PMOD	PMOD–QPET	QPET–SyngoMBF
Stress MBF	Global	0.91	0.84	0.86
	LAD	0.92	0.87	0.88
	LCX	0.90	0.81	0.84
	RCA	0.86	0.79	0.79
Rest MBF	Global	0.88	0.85	0.81
	LAD	0.87	0.85	0.84
	LCX	0.88	0.88	0.82
	RCA	0.81	0.80	0.69
MFR	Global	0.88	0.88	0.87
	LAD	0.88	0.89	0.88
	LCX	0.86	0.89	0.87
	RCA	0.80	0.77	0.69

**FIGURE 4:**
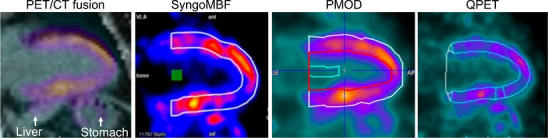
Representative images demonstrate that liver spillover affects automated placement of myocardial VOIs in the inferior wall. The leftmost panel shows a fused PET/CT image, with the liver and stomach indicated by white arrows. Automated VOIs include varying degrees of adjacent tissue. Therefore, manual correction is required for accurate myocardial delineation.

Figure 4 shows a situation in which the inferior myocardial wall was adjacent to the liver and stomach. The myocardial VOIs placed by all software tools shifted toward these adjacent structures, but the magnitude of displacement differed among them.

### 3.2 Effect of compartment model selection on quantitative indices

Table 4 shows global stress MBF, rest MBF, and MFR (mean ± SD) for 1TCM, Hutchins, and UCLA. All three indices differed among models (*P* < 0.05). Table 5 shows pairwise comparisons. Stress MBF differed in all paired models (*P* < 0.05). Rest MBF differed between 1TCM versus Hutchins and Hutchins versus UCLA, and MFR differed between Hutchins versus UCLA and UCLA versus 1TCM (*P* < 0.05). Stratified analyses by group (normal and CAD) showed similar results, although no significant difference in stress MBF was observed in the CAD group (Table S3).

**TABLE 4:** Global stress MBF, rest MBF, and MFR (mean ± SD) among compartment models (1TCM, Hutchins, UCLA).

	1TCM	Hutchins	UCLA	*P*. value
Stress MBF (mL/g/min)	2.33 ± 0.71	2.67 ± 0.94	3.03 ± 1.13	<0.05
Rest MBF (mL/g/min)	0.90 ± 0.17	1.09 ± 0.29	0.93 ± 0.22	<0.05
MFR	2.62 ± 0.80	2.56 ± 1.00	3.32 ± 1.22	<0.05

**TABLE 5:** Pairwise *P* values for global stress MBF, rest MBF, and MFR among compartment models (1TCM, Hutchins, UCLA).

	1TCM–Hutchins	Hutchins–UCLA	UCLA–1TCM
Stress MBF	<0.05	<0.05	<0.05
Rest MBF	<0.05	<0.05	0.62
MFR	0.91	<0.05	<0.05

**FIGURE 5:**
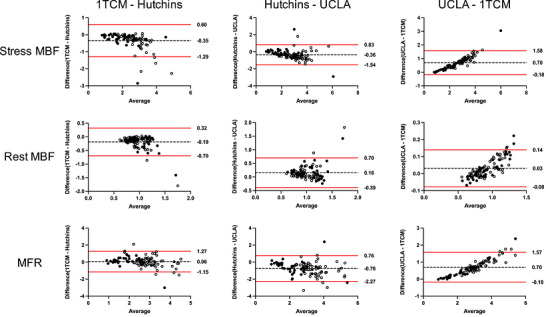
Bland–Altman plots of stress MBF, rest MBF, and MFR at global level compared among 1‐tissue compartment (1TCM), Hutchins, and UCLA models. The dashed lines indicate the mean difference, and the red solid lines represent the 95% limits of agreement. Open circles represent the Normal group, and filled circles represent the CAD group.

**FIGURE 6:**
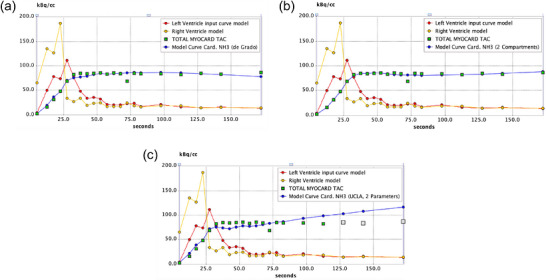
Myocardial time–activity curves (TACs) fitted by each compartment model in representative patient. (a) 1TCM, (b) Hutchins, (c) UCLA. Red and blue lines show input function from LV cavity and fitted myocardial TAC for each model.

Table 6 shows Pearson correlation coefficients (ρ) among compartment models at global and regional (LAD, LCX, RCA) levels. All model pairs demonstrated positive correlations at both global and regional levels. Values for ρ were lower for rest MBF in 1TCM versus Hutchins, and in Hutchins versus UCLA in all regions. Stratified analyses (normal and CAD groups; Table S4) showed that, consistent with Table 6, ρ values for rest MBF were lower for the 1TCM–Hutchins and Hutchins–UCLA comparisons than for other model pairs in both groups. Figure 5 shows Bland–Altman plots comparing MBF and MFR at the global level among the three compartment models, with the normal and CAD groups indicated by open and filled circles, respectively. Most values were within the 95% limits of agreement, and the degree of variability was similar between the two groups, indicating overall agreement. However, appreciable scatter remained, and a positive bias with the UCLA model was evident, particularly at higher values compared with 1TCM. Figure 6 shows representative time–activity curves (TACs) for each compartment model. The 1TCM and Hutchins models yield myocardial TACs that reach a plateau, whereas the UCLA model shows a continuous upward trend.

**TABLE 6:** Pearson correlation coefficients (ρ) for MBF and MFR among 1TCM, Hutchins, and UCLA compartment models at global and regional (LAD, LCX, RCA) levels.

		1TCM–Hutchins	Hutchins–UCLA	UCLA–1TCM
Stress MBF	Global	0.87	0.84	0.98
	LAD	0.89	0.86	0.98
	LCX	0.85	0.81	0.98
	RCA	0.84	0.84	0.98
Rest MBF	Global	0.47	0.43	0.97
	LAD	0.47	0.45	0.99
	LCX	0.61	0.58	0.99
	RCA	0.39	0.37	0.98
MFR	Global	0.79	0.77	0.99
	LAD	0.75	0.73	0.99
	LCX	0.80	0.76	0.98
	RCA	0.75	0.76	0.98

## 4 DISCUSSION

### 4.1 Inter‐software variability in MBF and MFR

The MBF and MFR showed high correlations among the software tools (Table 2, Figure 3) and agree with the findings of Slomka et al.^14^ based on earlier versions of QPET, SyngoMBF, and PMOD. The present study included more patients and was performed using a different PET/CT system and reconstruction method; however, the list‐mode acquisition methodology was consistent with that used in the study by Slomka et al.^14^ Even under these conditions, agreement among the software tools remained high, indicating overall agreement in MBF/MFR measurements. Stratified analysis by population (normal and CAD groups) showed generally similar trends between groups, although some differences were observed. Significant differences in stress MBF and MFR were identified only in the normal group, and relatively lower correlation coefficients for stress MBF were also observed in specific software pairs within this group. These findings suggest that while inter‐software agreement is broadly maintained, the magnitude of differences might vary depending on patient population. Although stress MBF statistically differed between PMOD and QPET (Table 2), the magnitude of the difference was limited.^14^ In both PMOD and QPET, VOIs are automatically placed and manually adjusted when needed; QPET applies software‐specific automated motion‐correction and VOI‐placement algorithms.^32^, ^33^, ^34^ PMOD differs in its use of dual spillover fractions (LV and RV). The compartment models also differed in that PMOD and QPET respectively adopt the methods of DeGrado et al.^22^ and Choi et al.^24^, ^35^ Reproducibility of MBF in ^82^Rb PET has been shown to decrease when different models are applied across software tools.^21^ These factors, including VOI definition, spillover handling, and model selection, might account for the differences identified herein. Inter‐software differences were more apparent under stress than at rest, probably because higher flow accentuates model‐ and processing‐dependent effects.^14^

Correlation coefficients for stress MBF, rest MBF, and MFR tended to be lower in the RCA territory than in the LAD and LCX (Table 3). This likely reflects the proximity of the inferior wall to the liver, where ^13^N‐ammonia uptake was high^31^ and resulted in frequent liver spillover to the inferior wall.^16^ Figure 4 shows that automated myocardial VOI settings might include adjacent extracardiac tissue when the heart is close to the liver, leading to overestimation and a need for manual correction. In addition, the fused PET/CT images suggest that the stomach might also contribute to this effect in some situations. The inferior wall is also susceptible to respiratory and body‐motion artifacts during PET acquisition, which can increase blood‐pool spillover.^14^, ^16^ Variation in the extent of manual correction and differences in spillover modeling among software tools likely contributed to the lower correlations in the RCA territory.^14^, ^16^

### 4.2 Effect of compartment model selection on quantitative indices

Previous studies of ^13^N‐ammonia PET have primarily focused on the theoretical formulation or validation of individual kinetic models. In contrast, the present study examined how commonly used kinetic models influence clinically reported MBF and MFR values when applied to the same clinical data under controlled analysis conditions. This perspective is clinically relevant, as MBF and MFR values are increasingly interpreted directly in routine practice. All three models differed in stress MBF (*P* < 0.05), and similar patterns were observed in stratified analyses (normal and CAD groups), although no significant difference in stress MBF was found in the CAD group. Only the Hutchins model differed in terms of rest MBF from the other two, and the UCLA model differed from the others in terms of MFR. The Hutchins model differed in both stress and rest MBF, with lower correlations in the latter. This relatively weaker correlation for rest MBF was consistently observed across both the normal and CAD groups. The Hutchins model estimates more parameters than the other models, which allows a closer approximation of tracer kinetics, but the additional degrees of freedom require attention to the precision and reproducibility of nonlinear least‐squares fitting.^37^ The findings in kinetic modeling with ^82^Rb are similar, as models with more parameters tend to have more inter‐subject variability.^21^, ^37^ The Hutchins model applied herein assessed five parameters and was the most complex of the three models. This might have influenced the fitting process and contributed to the differences.

The UCLA model generated higher values for stress MBF and MFR than 1TCM in particular. Derived from the Hutchins model, it fixes the distribution volume at 0.8 mL/g and expresses *k*2 and *k*3 as functions of *K*1.^24^, ^35^ Figure 6 shows that in contrast to the typical plateau behavior of myocardial TACs fitted by 1TCM and Hutchins, the UCLA model produced a continuous upward trend in myocardial activity, reflecting model‐specific parameter constraints to the myocardial compartment. This might have contributed to higher myocardial perfusion estimates with the UCLA model, particularly at higher values. Quantitation of MBF and MFR is also performed using other modalities, such as cadmium‐zinc‐telluride (CZT)‐based SPECT systems. Similar methodological challenges, including tracer kinetics, spillover correction, and model selection, have been reported in these modalities.^38^, ^39^ Differences in kinetic modeling and analytical implementations can influence MBF and MFR estimates in these systems. Although the present study focused on ^13^N‐ammonia PET, the findings regarding variability related to analytical methods might also be relevant to these alternative imaging approaches.

This study has several limitations. The absence of an independent reference standard precluded determination of which kinetic model most closely reflects true MBF. The present findings therefore demonstrate model‐dependent variability rather than preferential performance of any specific approach. The individual contributions of VOI placement, degree of manual correction, spillover‐fraction handling, and kinetic modeling assumptions were not independently assessed. Spillover from adjacent extracardiac organs, such as the liver and stomach, might have contributed to lower correlations in the RCA territory, and fully automated estimation of spillover fractions for such interference remains technically challenging. In addition, the Hutchins implementation incorporated the proprietary metabolite correction in PMOD, and its impact on MBF estimates was not separately evaluated. Furthermore, some differences were observed between the normal and CAD groups, and further investigation is required to determine whether these differences are attributable to underlying pathophysiology or other factors.

## 5 CONCLUSIONS

The MBF and MFR calculated using three software tools closely correlated at the global and regional levels, and MFR values showed high correlation across software tools. These findings indicate that MBF/MFR quantitation under standardized imaging and analysis shows overall agreement among different software tools, although residual variability remains. In contrast, compartment‐model selection considerably impacted MBF and MFR. The UCLA two‐tissue model consistently yielded higher values for both indices than 1TCM and the Hutchins model. The choice of model is therefore critical for ^13^N‐ammonia PET perfusion quantitation and can meaningfully influence the interpretation of absolute perfusion values.

## AUTHOR CONTRIBUTIONS

**Naochika Akiya**, **Kenta Miwa**, **Akira Ando**, and **Noriaki Miyaji** designed the study. AA collected the data. NA and AA processed the data. Naochika Akiya, Kenta Miwa, Akira Ando, Noriaki Miyaji, **Tensho Yamao**, **Kaito Wachi**, **Masaki Masubuchi**, **Shu Kimura**, **Reiji Ito**, **Arata Komatsu**, **Takuma Horikawa** and **Kenji Fukushima** interpreted the data. Naochika Akiya, Kenta Miwa, and Noriaki Miyaji drafted and revised the manuscript. All authors read and approved the final version of the manuscript.

## CONFLICT OF INTEREST STATEMENT

The authors declare that they have no competing interests.

## ETHICAL STATEMENT

The Ethics Committee of Sapporo Kojinkai Memorial Hospital approved this study of 100 patients who were assessed by ^13^N‐ammonia PET/CT at Sapporo Kojinkai Memorial Hospital (Approval no: 2024‐6). All patients provided written informed consent to participate in the study, which complied with the ethical principles enshrined in the Declaration of Helsinki (2013 amendment).

## Supporting information




**Supporting Information**: acm270605‐supp‐0001‐SuppMat.docx

**Supporting Information**: acm270605‐supp‐0002‐SuppMat.docx

**Supporting Information**: acm270605‐supp‐0003‐SuppMat.docx

**Supporting Information**: acm270605‐supp‐0004‐SuppMat.docx

## Data Availability

The data that support the findings of this study are not publicly available due to institutional and ethical restrictions regarding patient confidentiality but are available from the corresponding author upon reasonable request and with appropriate institutional approval.
