# Supplementary material for: Impact of software tools and kinetic model selection on myocardial blood flow and flow reserve quantitation in 13N‐ammonia PET
Source: J Appl Clin Med Phys. 2026 May 1;27(5):e70605. doi: 10.1002/acm2.70605 (PMC13134436; doi:10.1002/acm2.70605)
Supplement: Supplementary file 1 — Supporting Information: acm270605‐supp‐0001‐SuppMat.docx [file ACM2-27-e70605-s002.docx]

Table S1. Global stress MBF, rest MBF, and MFR (mean ± SD) stratified by population (normal and CAD) using SyngoMBF, PMOD, and QPET software tools.

|  | Population | SyngoMBF | PMOD | QPET | p value |
| --- | --- | --- | --- | --- | --- |
| Stress MBF (mL/g/min) | Normal (n=60) | 2.85 ± 0.53 | 2.66 ± 0.48 | 3.02 ± 0.43 | <0.05 |
|  | CAD (n=40) | 1.77 ± 0.71 | 1.83 ± 0.71 | 1.95 ± 0.66 | 0.49 |
| Rest MBF (mL/g/min) | Normal (n=60) | 0.94 ± 0.19 | 0.90 ± 0.16 | 0.92 ± 0.23 | 0.50 |
|  | CAD (n=40) | 0.92 ± 0.21 | 0.91 ± 0.19 | 0.95 ± 0.26 | 0.72 |
| MFR | Normal (n=60) | 3.17 ± 0.63 | 3.01 ± 0.60 | 3.44 ± 0.74 | <0.05 |
|  | CAD (n=40) | 1.98 ± 0.71 | 2.03 ± 0.70 | 2.12 ± 0.71 | 0.66 |
