# Supplementary material for: Impact of software tools and kinetic model selection on myocardial blood flow and flow reserve quantitation in 13N‐ammonia PET
Source: J Appl Clin Med Phys. 2026 May 1;27(5):e70605. doi: 10.1002/acm2.70605 (PMC13134436; doi:10.1002/acm2.70605)
Supplement: Supplementary file 2 — Supporting Information: acm270605‐supp‐0002‐SuppMat.docx [file ACM2-27-e70605-s003.docx]

Table S2. Pearson correlation coefficients (ρ) for MBF and MFR among software at the global, stratified by population (normal and CAD).

|  | Population | SyngoMBF–PMOD | PMOD–QPET | QPET–SyngoMBF |
| --- | --- | --- | --- | --- |
| Stress MBF | Normal (n=60) | 0.77 | 0.58 | 0.53 |
|  | CAD (n=40) | 0.94 | 0.87 | 0.89 |
| Rest MBF | Normal (n=60) | 0.90 | 0.86 | 0.82 |
|  | CAD (n=40) | 0.86 | 0.84 | 0.81 |
| MFR | Normal (n=60) | 0.71 | 0.76 | 0.71 |
|  | CAD (n=40) | 0.92 | 0.88 | 0.85 |
