# Supplementary material for: Impact of software tools and kinetic model selection on myocardial blood flow and flow reserve quantitation in 13N‐ammonia PET
Source: J Appl Clin Med Phys. 2026 May 1;27(5):e70605. doi: 10.1002/acm2.70605 (PMC13134436; doi:10.1002/acm2.70605)
Supplement: Supplementary file 3 — Supporting Information: acm270605‐supp‐0003‐SuppMat.docx [file ACM2-27-e70605-s001.docx]

Table S3. Global stress MBF, rest MBF, and MFR (mean ± SD) stratified by population (normal and CAD) among compartment models (1TCM, Hutchins, UCLA).

|  | Population | 1TCM | Hutchins | UCLA | p value |
| --- | --- | --- | --- | --- | --- |
| Stress MBF (mL/g/min) | Normal (n=60) | 2.66 ± 0.48 | 3.08 ± 0.78 | 3.53 ± 0.75 | <0.05 |
|  | CAD (n=40) | 1.83 ± 0.71 | 2.07 ± 0.84 | 2.27 ± 1.18 | 0.12 |
| Rest MBF (mL/g/min) | Normal (n=60) | 0.90 ± 0.16 | 1.06 ± 0.28 | 0.93 ± 0.20 | <0.05 |
|  | CAD (n=40) | 0.91 ± 0.19 | 1.13 ± 0.30 | 0.94 ± 0.25 | <0.05 |
| MFR | Normal (n=60) | 3.01 ± 0.60 | 3.00 ± 0.80 | 3.89 ± 0.92 | <0.05 |
|  | CAD (n=40) | 2.03 ± 0.70 | 1.90 ± 0.91 | 2.45 ± 1.09 | <0.05 |
