# Supplementary material for: Impact of software tools and kinetic model selection on myocardial blood flow and flow reserve quantitation in 13N‐ammonia PET
Source: J Appl Clin Med Phys. 2026 May 1;27(5):e70605. doi: 10.1002/acm2.70605 (PMC13134436; doi:10.1002/acm2.70605)
Supplement: Supplementary file 4 — Supporting Information: acm270605‐supp‐0004‐SuppMat.docx [file ACM2-27-e70605-s004.docx]

Table S4. Pearson correlation coefficients (ρ) for MBF and MFR among 1TCM, Hutchins, and UCLA compartment models at global, stratified by population (normal and CAD).

|  | Population | 1TCM–Hutchins | Hutchins–UCLA | UCLA–1TCM |
| --- | --- | --- | --- | --- |
| Stress MBF | Normal (n=60) | 0.80 | 0.77 | 0.96 |
|  | CAD (n=40) | 0.84 | 0.81 | 0.98 |
| Rest MBF | Normal (n=60) | 0.40 | 0.37 | 0.98 |
|  | CAD (n=40) | 0.55 | 0.50 | 0.99 |
| MFR | Normal (n=60) | 0.65 | 0.61 | 0.97 |
|  | CAD (n=40) | 0.73 | 0.73 | 0.99 |
